# Jalili Syndrome: Cross-sectional and Longitudinal Features of Seven Patients With Cone-Rod Dystrophy and Amelogenesis Imperfecta

**DOI:** 10.1016/j.ajo.2018.01.029

**Published:** 2018-04

**Authors:** Nashila Hirji, Patrick D. Bradley, Shuning Li, Ajoy Vincent, Mark E. Pennesi, Akshay S. Thomas, Elise Heon, Aparna Bhan, Omar A. Mahroo, Anthony Robson, Chris F. Inglehearn, Anthony T. Moore, Michel Michaelides

**Affiliations:** a Moorfields Eye Hospital NHS Foundation Trust and UCL Institute of Ophthalmology, University College London, London, United Kingdom; b Genetics and Genome Biology, Hospital for Sick Children, Toronto, Canada; c Department of Ophthalmology and Vision Sciences, Hospital for Sick Children, University of Toronto, Toronto, Canada; d Oregon Health & Science University, Casey Eye Institute, Portland, Oregon; e Duke University Eye Center, Durham, North Carolina; f School of Medicine, University of Leeds, United Kingdom; g UCSF School of Medicine, Department of Ophthalmology, San Francisco, California

## Abstract

**Purpose:**

To characterize a series of 7 patients with cone-rod dystrophy (CORD) and amelogenesis imperfecta (AI) owing to confirmed mutations in *CNNM4*, first described as “Jalili Syndrome.”

**Design:**

Retrospective observational case series.

**Methods:**

Seven patients from 6 families with Jalili Syndrome were identified at 3 tertiary referral centers. We systematically reviewed their available medical records, spectral-domain optical coherence tomography (SD-OCT), fundus autofluorescence imaging (FAF), color fundus photography, and electrophysiological assessments.

**Results:**

The mean age at presentation was 6.7 years (range 3-16 years), with 6 male and 1 female patient. *CNNM4* mutations were identified in all patients. The mean Snellen best-corrected visual acuity (BCVA) at presentation was 20/246 (range 20/98 to 20/399) in the right eye and 20/252 (range 20/98 to 20/480) in the left. Nystagmus was observed in all 7 patients, and photophobia was present in 6. Funduscopic findings at presentation were variable, ranging from only mild disc pallor to retinal vascular attenuation and macular atrophy. Multimodal imaging demonstrated disease progression in all 7 patients over time. Electroretinography uniformly revealed progressive cone-rod dysfunction.

**Conclusions:**

Jalili Syndrome is a rare CORD associated with AI. We have further characterized its ocular phenotype, including describing SD-OCT, FAF, and electrophysiological features; and report several novel disease-causing sequence variants. Moreover, this study presents novel longitudinal data demonstrating structural and functional progression over time, allowing better informed advice on prognosis.

The association of amelogenesis imperfecta (AI) with cone-rod dystrophy (CORD) was originally described in a consanguineous Arabic family with 29 affected members, who resided in the Gaza strip.^1^, ^2^, ^3^ The condition has been given the eponym Jalili syndrome, after one of the authors who originally described the condition in 1988.^3^

The CORDs are a group of inherited retinal disorders that demonstrate greater cone than rod dysfunction, reduced visual acuity, photophobia, poor color vision, and, in later stages, nyctalopia and peripheral visual field constriction.^2^, ^4^ A minority of CORDs have additional systemic associations.^4^, ^5^, ^6^ One of the primary features of Jalili syndrome is AI.^1^, ^2^, ^3^ AI is a hereditary group of disorders affecting the deposition of tooth enamel. The condition can affect both the composition and the structure of tooth enamel, and can broadly be classified into 2 groups. The first group demonstrates a quantitative defect of enamel, and is known as primarily hypoplastic AI. This is characterized by thin or missing areas of enamel, but the teeth are otherwise normal in structure. The second group is defined by a qualitative defect of enamel, and is known as the hypomature or hypomineralized variant of AI.^2^, ^7^, ^8^, ^9^

The original Gaza family with Jalili syndrome demonstrated genetic linkage to 2q11.^2^, ^3^, ^10^ In our previous publication describing 2 Kosovan brothers with this condition, we also established linkage to 2q11.^2^ Subsequently, Parry and associates identified 9 disease-causing variants in *CNNM4* (encoding a putative metal transporter) in these brothers and other affected patients (from Gaza, Scotland, Turkey, Guatemala, and Iran).^3^

The purpose of our study was to clinically characterize 7 patients from 6 families with genetically confirmed Jalili syndrome. We describe the ocular phenotype, including a description of spectral-domain optical coherence tomography (SD-OCT), fundus autofluorescence imaging (FAF), electrophysiological features, and visual parameters; and present novel longitudinal data for all patients, 2 of whom have more than 15 years of follow-up at the same center. We identify that Jalili syndrome is characterized by a severe early-onset and progressive CORD, with a poor visual prognosis.

## Methods

In this retrospective multicenter observational study, clinical data including best-corrected Snellen acuity, color fundus photography, SD-OCT and FAF imaging, and electrophysiological assessments were reviewed for 7 affected patients. Four patients with disease-causing variants in *CNNM4* were identified at Moorfields Eye Hospital (London, United Kingdom), 1 at Casey Eye Institute (Portland, Oregon, USA), and 2 at the Hospital for Sick Children, Toronto (Canada).

SD-OCT and FAF images were obtained with a Spectralis (Heidelberg Engineering, Heidelberg, Germany) or Cirrus SD-OCT (Carl Zeiss Meditec, Dublin, California, USA) and Visucam Carl Zeiss Meditec AG, Jena, Germany. Approval for data collection and analysis was obtained from the Institutional Review Boards of Moorfields Eye Hospital, Casey Eye Institute, and the Hospital for Sick Children, Toronto. The study adhered to the tenets set forth in the Declaration of Helsinki.

## Results

### Patient Characteristics

**Figure 1:**
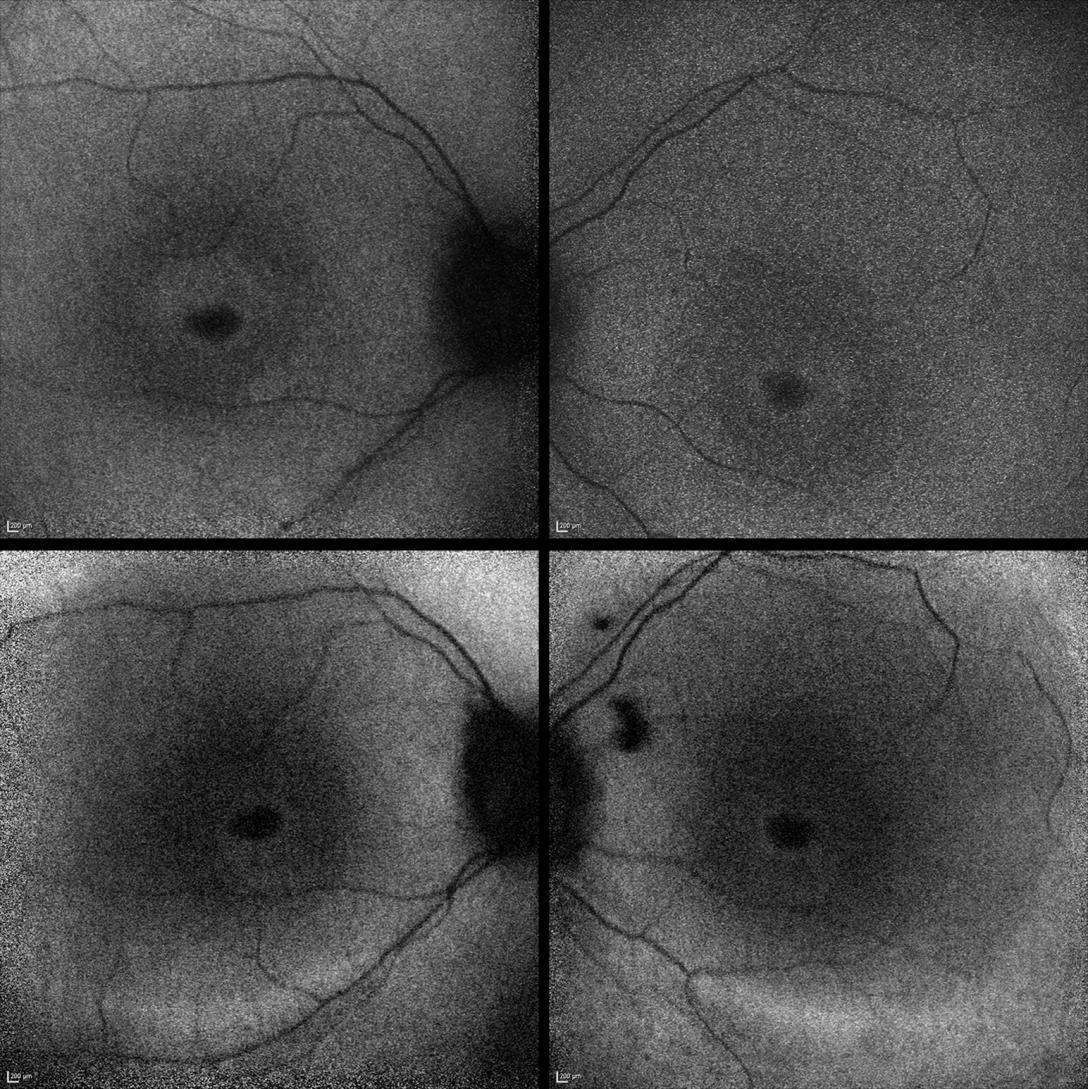
Fundus autofluorescence imaging of Patient 3. (Top left) Perifoveal ring of increased autofluorescence in the right eye. (Top right) Similar findings in the left eye. Repeat imaging 5 years later demonstrated a significant increase in the size of the rings, in both the right eye (Bottom left) and left eye (Bottom right).

**Figure 2:**
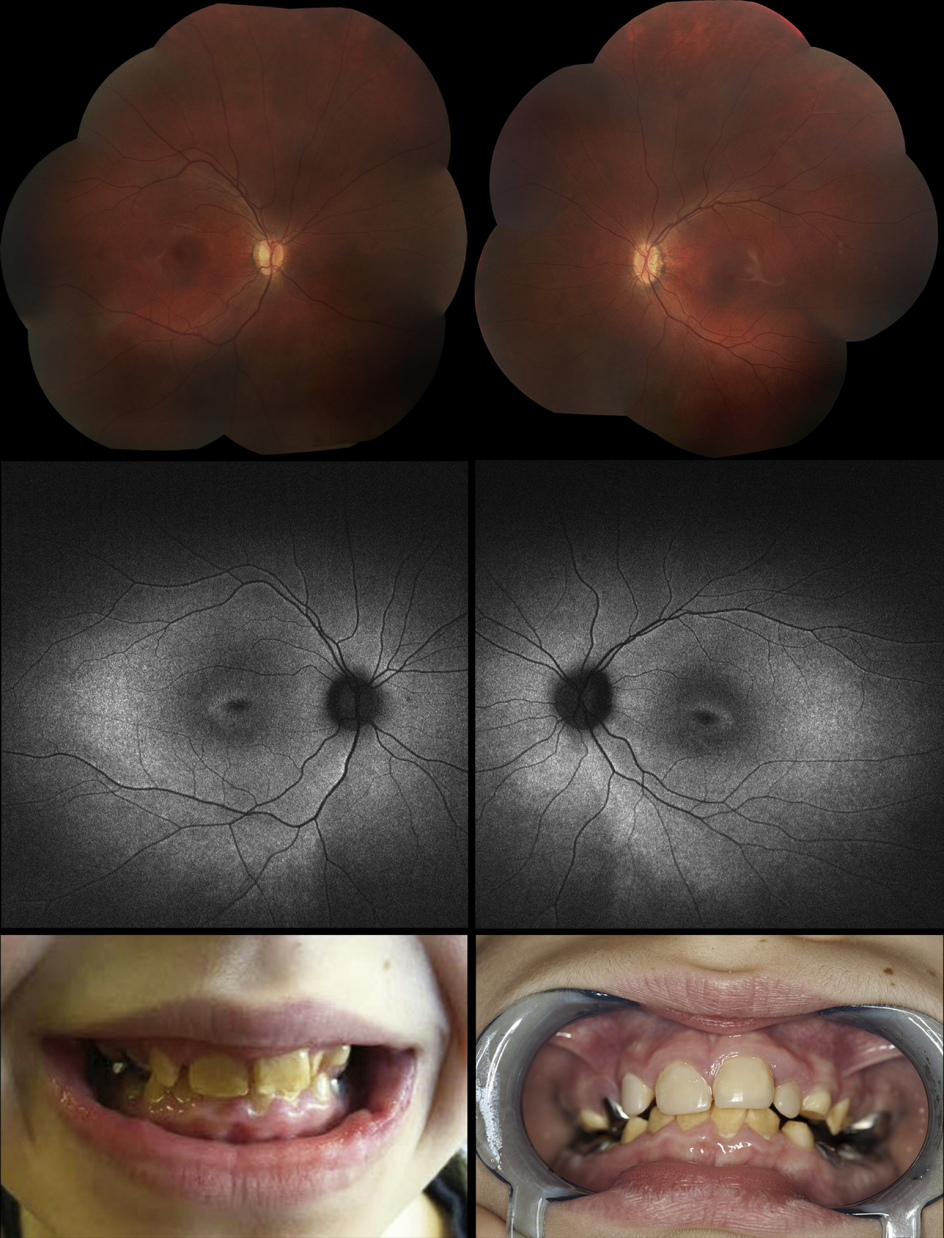
Images demonstrating the phenotype of Patient 5. Fundus appearances at presentation showing tilted optic discs, loss of the foveal light reflex, and mild vascular attenuation in the right eye (Top left) and left eye (Top right). Fundus autofluorescence imaging 3 years later showed generalized hyperautofluorescence in the posterior pole as well as a ring of hyperautofluorescence around the fovea in both the right (Middle left) and left (Middle right) eyes. (Bottom left) Teeth at presentation, demonstrating enamel hypoplasia and the presence of crowns. (Bottom right) Teeth 3 years later, after upper teeth had been resurfaced.

**Figure 3:**
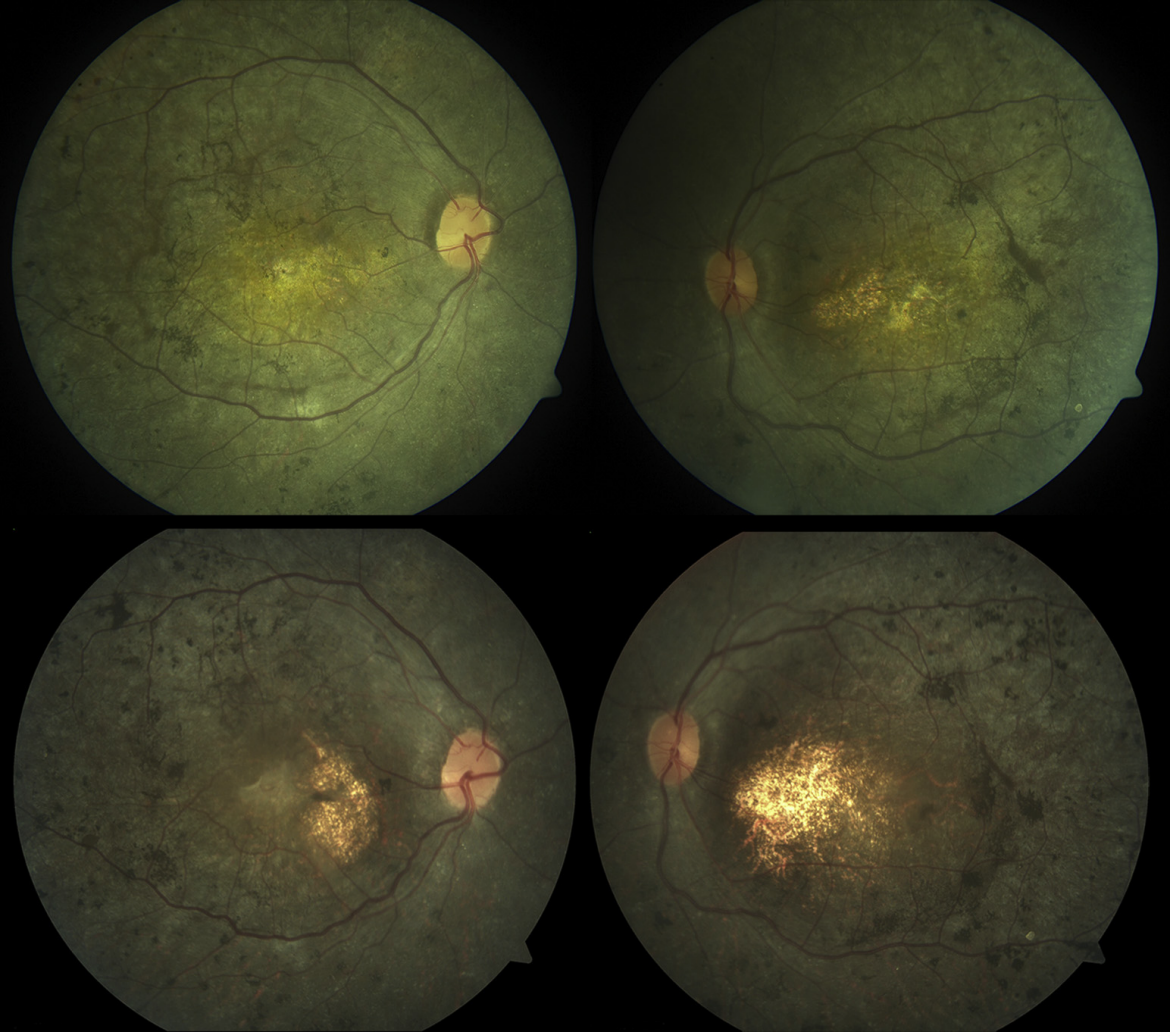
Color fundus images of Patient 7. Bilateral macular atrophy with retinal pigment migration, in the right eye (Top left) and left eye (Top right), at age 22 years. Repeat imaging 5 years later demonstrated advancement of pigmentary changes and increased macular atrophy in both the right eye (Bottom left) and left eye (Bottom right).

Of the 7 patients included in our study, 6 were male, with a mean age at presentation of 6.7 years (range 3-16 years). The mean Snellen best-corrected visual acuity (BCVA) at the time of presentation was 20/246 (range 20/98 to 20/399) in the right eye and 20/252 (range 20/98 to 20/480) in the left. All 7 patients had nystagmus at presentation, with 6 having photophobia. All patients demonstrated features consistent with AI on dental examination, with 2 patients specifically diagnosed with the hypoplastic variant (Patients 1 and 2). Patient characteristics are summarized in the Table and shown in Figure 1, Figure 2, Figure 3.

**Table:** Summary of Clinical Features of Jalili Syndrome Patients

Patient No.	Source	Sex	Ethnicity	Age at Presentation (Years)	Follow-up Duration (Years)	Nystagmus	Photophobia	BCVA at Presentation (Snellen)		BCVA at Final Follow-up (Snellen)		*CNNM4* Mutations		Novel/Previously Reported
								OD	OS	OD	OS	Variant 1	Variant 2	
1	Moorfields Eye Hospital	M	Kosovan	6	15	+	+	20/399	20/399	HM	HM	c.1312 dupC; p.Leu438Profs*9	c.1312 dupC; p.Leu438Profs*9	Previously reported^3^
2	Moorfields Eye Hospital	M	Kosovan	4	15	+	+	20/399	20/399	CF	CF	c.1312 dupC; p.Leu438Profs*9	c.1312 dupC; p.Leu438Profs*9	Previously reported^3^
3	Moorfields Eye Hospital	M	Kosovan	5	10	+	+	20/200	20/200	20/126	20/126	c.1312 dupC; p.Leu438Profs*9	c.1312 dupC; p.Leu438Profs*9	Previously reported^3^
4	Moorfields Eye Hospital	M	Pakistani	3	2.5	+	+	20/98	20/98	20/200	20/252	c.1226C>T; p.Pro409Leu	c.1226C>T; p.Pro409Leu	Novel
5	Casey Eye Institute	M	English/ Irish/ German	6	4	+	+	20/246	20/200	20/200	20/200	c.1307delC; p.Leu438Serfs*41	c.C1690T; p.Gln564*	Variant 1: Novel Variant 2: Previously reported^3^
6	University of Toronto	F	Afghani	7	38	+	+	20/200	20/200	PL	PL	c.C734T; p.Ser245Leu	c.C734T; p.Ser245Leu	Novel
7	University of Toronto	M	Afghani	16	11	+	-	20/317	20/480	20/1002	20/796	c.C734T; p.Ser245Leu	c.C734T; p.Ser245Leu	Novel

CF= counting fingers; HM = hand motion; PL = perception of light.

### Patients 1 and 2

Patients 1 and 2 in our study were brothers of Kosovan descent and have been briefly described previously.^2^ They presented to Moorfields Eye Hospital at ages 6 and 4, respectively, with fine pendular nystagmus, reduced visual acuity, and marked photophobia from early infancy—raising the clinical suspicion of achromatopsia. There was no family history of consanguinity. Initial BCVA was 20/399 bilaterally in each patient, with a hypermetropic and astigmatic correction. Fundus examination revealed bilateral macular atrophy in both siblings. Both brothers described difficulty with night vision 5-6 years after presentation. At final review, 15 years after presentation, Patient 1 had hand motion vision in both eyes and Patient 2 had counting fingers vision bilaterally.

Both brothers underwent detailed electrophysiological assessment at presentation, including full-field electroretinogram (ERG), pattern ERG (PERG), and electro-oculogram. This revealed no detectable cone ERGs, with markedly reduced rod function in both patients. Repeat ERGs 12 years later demonstrated progressive deterioration of retinal function over time; no definite responses could be demonstrated from either eye under any stimulus or recording conditions in Patient 1, and only a very low-amplitude response with a bright flash under dark adaptation was recorded in Patient 2.

Detailed color vision testing of the 2 affected brothers and their parents was performed early in the course of their evaluation. This included the use of the Hardy, Rand, and Rittler plates (American Optical Company, New York, New York, USA), Farnsworth Munsell (FM) 100 hue test, Farnsworth D-15, the Mollon-Reffin (MR) minimal test, a computerized color vision test, and anomaloscopy.^2^ The testing failed to demonstrate any residual color vision in either patient in childhood.

Both siblings had serial OCT performed over a period of 12 years. They demonstrated profound loss of outer retinal architecture in their initial images, with evidence of progressive loss of photoreceptor layers over time. In parallel, progression of macular atrophy was noted clinically and on FAF imaging in both patients.

In addition, both siblings demonstrated features consistent with the hypoplastic variant of AI, which was diagnosed on extensive dental evaluations at an early age.^2^

Both patients were found to be homozygous for the c.1312 dupC; p.Leu438Profs*9 variant in *CNNM4.*^3^

### Patient 3

Patient 3 presented to Moorfields Eye Hospital at age 5 with decreased visual acuity (20/200 in both eyes), nystagmus, and photophobia. He was of Kosovan descent, and there was no family history of consanguinity. BCVA at follow-up 10 years later was 20/126 bilaterally.

ERG testing at presentation, performed using skin electrodes, revealed findings in keeping with a cone-rod dystrophy with severe macular involvement bilaterally. Repeat ERG testing with gold foil electrodes 5 years later demonstrated undetectable PERGs, rod ERGs, and single flash cone ERGs, with a deterioration in the bright flash ERG.

Recent SD-OCT revealed a profound loss of outer retinal architecture at the central macula in both eyes. Initial FAF imaging at age 9 years demonstrated a perifoveal ring of hyperautofluorescence in both eyes; repeat imaging 5 years later revealed a significant increase in the size of the rings bilaterally (Figure 1).

The patient was found to be homozygous for the c.1312 dupC; p.Leu438Profs*9 *CNNM4* variant.

### Patient 4

Patient 4 presented to Moorfields Eye Hospital at age 3 with a history of decreased vision, difficulty navigating during the day, photophobia, and nystagmus. He was of Pakistani descent, and his parents were consanguineous. Initial visual acuity was 20/98 bilaterally, and retinal examination was essentially normal, although it was noted that his optic discs appeared slightly pale. Electrophysiological assessment performed at presentation with skin electrodes was limited by patient cooperation, but showed that no consistent retinal responses were evident for either eye to mixed rod-cone stimuli. Pattern-reversal visual evoked potentials were not evident. At follow-up 2.5 years later, BCVA was 20/200 in the right eye, and 20/252 in the left.

He was found to be homozygous for the c.1226C>T; p.Pro409Leu sequence variant in *CNNM4.*

### Patient 5

Patient 5 presented to Casey Eye Institute at 6 years of age for evaluation of nystagmus. He had a combination of English, Irish, and German ancestry. There was no family history of consanguinity. He was noted to have decreased visual acuity and nystagmus since birth, and photophobia during his first year of life. The patient had a history of enamel hypoplasia, for which he had had multiple dental extractions and crowns placed (Figure 2, Bottom, left and right). At presentation, BCVA was 20/246 in the right eye and 20/200 in the left. Fundus examination showed tilted optic discs, loss of the foveal light reflex, and mild vascular attenuation bilaterally (Figure 2, Top, left and right).

At follow-up 3 years later, kinetic perimetry showed normal responses to the V4e and III4e isopters but constriction to the I4e, I3e, and I2e isopters bilaterally, as well as enlarged blind spots. SD-OCT demonstrated severe outer retinal atrophy bilaterally. FAF imaging showed perivascular hyperautofluorescence in the posterior pole as well as a distinct parafoveal ring of hyperautofluorescence surrounding a hypoautofluorescent fovea (Figure 2, Middle, left and right).

Multifocal ERG revealed unrecordable macular cone responses in both eyes. Full-field ERG demonstrated normal DA0.01 recordings but reduction of both the b- and a-wave to the DA6.0 response. Cone-driven responses (both LA6.0 and 30 Hz flicker) were severely reduced in each eye.

At his last follow-up visit, 4 years after presentation, BCVA was 20/200 bilaterally.

He was found to harbor compound heterozygous variants in *CNNM4*, c.1307delC; p.Leu438Serfs*41 and c.C1690T; p.Gln564*.

### Patient 6

Patient 6, born to consanguineous parents of Afghani origin, was recently examined at The Hospital for Sick Children at age 45. She had photophobia since early childhood and nystagmus observed at age 7. Her BCVA at 7 years of age was 20/200 bilaterally. On review 37 years later, BCVA was light perception in both eyes, with fundus examination revealing pale discs, severe macular atrophy, attenuated vessels, and peripheral retinal pigmentary changes bilaterally. SD-OCT demonstrated extremely disrupted central retinal layers with evidence of traction and foveal schisis in both eyes. FAF revealed marked hypoautofluorescence centrally.

Her dental history was in keeping with a diagnosis of AI. The patient's milk teeth began developing at 7 months of age and were noted to be yellowish in color at the time. Her permanent teeth, which also had yellowish coloration, were all removed and replaced with artificial dentition at 26 years of age.

She was found to be homozygous for the c.C734T; p.Ser245Leu variant in *CNNM4.*

### Patient 7

Patient 7 presented to the University of Toronto at age 16, with symptoms of nyctalopia and poor vision since infancy. He was born to consanguineous parents of Afghani origin and was the cousin of Patient 6. At his initial evaluation, he had pendular nystagmus. The BCVA was 20/317 and 20/480 in the right and left eyes, respectively. Goldmann visual field testing was constricted to the central 20 degrees to the IV4e target. Full-field ERG showed nondetectable rod and cone responses. Fundus examination showed bilateral macular atrophy with scalloped patchy deep retinal atrophy outside the arcades.

At follow-up evaluation at age 27, BCVA was 20/1002 and 20/796 in the right and left eyes, respectively. There was evidence of progressive visual field loss on Goldmann perimetry (binocular central fields of 5 degrees to IV4e target). Progressive macular atrophy was observed clinically and on SD-OCT, with additional peripheral retinal pigment migration in the periphery since last review (Figure 3).

The patient was found to be homozygous for the c.C734T; p.Ser245Leu variant in *CNNM4.*

## Discussion

Jalili syndrome is a rare autosomal recessive condition, comprising CORD in association with AI. Ocular symptoms reflecting predominantly cone photoreceptor dysfunction usually manifest in early childhood/infancy with photophobia, nystagmus, reduced visual acuity, and color vision defects. Nyctalopia and visual field defects may develop later, reflecting rod photoreceptor involvement.^2^, ^4^ AI encompasses a range of genetically heterogeneous conditions that affect dental development. It results in abnormal structure and appearance of tooth enamel, in almost all the teeth of both primary and secondary dentition. There is discoloration, sensitivity, and brittleness of teeth, with premature tooth loss.^11^ As well as occurring in conjunction with CORD in Jalili syndrome, AI can also occur in isolation, or within syndromes in which a wider range of tissues and organs are affected.^12^

Jalili syndrome has been described in 29 families and 108 patients worldwide.^13^ It shows phenotypic diversity within and between affected families.^14^, ^15^ The condition was linked to chromosome 2q11^2^, ^3^ and later mutations in the cyclin M4 (*CNNM4*) gene were identified as the underlying cause of the condition.^3^, ^16^

The protein product of *CNNM4* is expressed in the retina, and has been implicated in the transport of metal ions, in particular magnesium.^17^ Magnesium is thought to be essential for the normal function of photoreceptors and for homeostasis within the retina.^18^ In addition, *CNNM4* expression has been demonstrated in tooth enamel and dentin-forming cells, the ameloblasts and odontoblasts, suggesting that abnormal magnesium transport can lead to the hypomineralization of dental enamel seen in AI.^3^, ^16^

To date, 20 mutations have been reported in *CNNM4*, in association with Jalili syndrome.^13^ The mutations include missense, nonsense, large deletions, single base insertion, and duplication. They often occur within the conserved domains of the protein, resulting in loss of function.^19^ Of note, in our patients we report several likely disease-causing novel *CNNM4* mutations (Table). Patient 4 was found to be homozygous for the c.1226C>T; p.Pro409Leu variant in *CNNM4.* This mutation is novel. Proline at codon 409 is highly conserved, and this substitution is predicted to be damaging. Patient 5 harbored compound heterozygous mutations in *CNNM4*: c.1307delC; p.Leu438Serfs*41 (a novel frameshift mutation predicted to be deleterious) and c.C1690T; p.Gln564* (a previously reported nonsense mutation).^3^ Patients 6 and 7 were found to be homozygous for the novel c.C734T; p.Ser245Leu variant in *CNNM4.* The amino acid affected is highly conserved among mammals and vertebrates and is predicted to be pathogenic.

Rahimi-Aliabadi and associates recently described the detailed ocular and dental phenotype in a large consanguineous Iranian pedigree with Jalili syndrome, including 24 affected members from a 7-generation family.^20^ Though the group described the variability in ocular phenotype, there remains a lack of published data on the natural history of this condition. To address this, in addition to elucidating the phenotype and clinical characteristics of 7 patients with molecularly confirmed Jalili syndrome, we present longitudinal data on the largest cohort of affected individuals to date.

In our series, each patient presented with reduced acuity and nystagmus in infancy, with evidence of cone or cone-rod dystrophy on ERG. Fundus appearance varied between patients, ranging from macular atrophy with pigmentary changes to a normal retinal appearance apart from mild optic disc pallor; much of this variation reflects the age at examination, with older patients showing more severe retinal changes. In parallel to fundus findings, FAF appearances showed change over time, and serial SD-OCT, though often limited in quality owing to the subjects' photophobia and nystagmus, showed progressive deterioration of central retinal architecture. In summary, our analysis of the clinical data available for each patient demonstrates a worsening in retinal structure and function over the years.

One of the inherent weaknesses of our study is the retrospective nature of the case series and the small sample size, although this is perhaps inevitable with such rare disorders. Our most valuable findings include that patients harboring disease-causing variants in *CNNM4* suffer from an early-onset severe and progressive CORD, in conjunction with AI, with the visual prognosis for these patients being poor. It is therefore important that clinicians recognize Jalili syndrome early so that patients can receive appropriate support for their visual impairment, advice on prognosis, genetic counseling, and the dental interventions that they require.
